# Concept Evolution and Multi-Dimensional Measurement Comparison of Urban Energy Performance from the Perspective of System Correlation: Empirical Analysis of 142 Prefecture Level Cities in China

**DOI:** 10.3390/ijerph182413046

**Published:** 2021-12-10

**Authors:** Lei Wang, Wei Li, Guomin Li, Guozhen Zhang

**Affiliations:** School of Economics and Management, Taiyuan University of Technology, Taiyuan 030024, China; liwei01@tyut.edu.cn (W.L.); liguomin@tyut.edu.cn (G.L.); zhangguozhen@tyut.edu.cn (G.Z.)

**Keywords:** system association, urban energy performance, concept evolution, multi-dimensional measurement comparison

## Abstract

In order to clarify the evolution characteristics and direction of urban energy performance concepts, reveal the research dimensions, determine the performance results and differences, and clarify the reference benchmark, this study depicts the main systems involved in the process of urban energy utilization, demonstrates their relevance guided by the system view, and proposes the measurement indicators in the economic, environmental, and well-being dimensions. The measurement model of each dimension is constructed using the corresponding models of Data Envelopment Analysis. Taking 142 prefecture level cities in China as examples, the energy performance in different dimensions is measured and compared. The energy performance levels are close in the economic and environmental dimensions. However, the results of the well-being dimension are different from these first two dimensions, and the performance levels among cities differ more. In the economic, environmental, and well-being dimension, 22, 28 and 16 cities have reached the effective frontier, respectively, and the performance benchmark cities of 15, 15 and 5 provinces are non-provincial capital cities, respectively. Based on the above analysis, the “chain” framework evolution direction of concept and measurement is proposed, and this study provides benchmarks and policy suggestions to improve energy performance.

## 1. Introduction

As the mainstay of urban development, energy provides great power for urban production and societal activity. With the rapid advancement of industrialization, urban economy has achieved rapid growth [1]. However, along with economic growth, there are problems such as ineffective energy utilization and excessive energy consumption, resulting in insufficient energy supply and a decline in environmental quality [2]. According to the statistics, although the urban area accounts for only 2% of the world’s total area, the energy consumption and the greenhouse gas emissions of cities have accounted for about 75% and 80% of the world’s energy consumption and greenhouse gas emissions, respectively [3]. China’s population weighted average PM2.5 concentration ranked first among the world’s most populous countries, and has increased significantly from 1990 to 2010 [4]. In 2017, less than one third of China’s prefecture level cities met the air quality standards [5]. The pollution is more serious in winter, as there is a black and odorous water body near the city. Traditional and new environmental problems occur frequently [6,7,8].

As a result of urban production and living, and industrial and traffic environmental pollution [9], cities will face greater pressure in ecological construction and sustainable development. The safety, health, life satisfaction, life quality and well-being of urban residents [10] have gradually become the focus of attention [11]. The Global Air State Report of 2019 pointed out that about five million people died of air pollution worldwide and China’s mortality rate ranked among the top 10 countries in the world in 2017 [12]. It can be evidenced that urban energy is closely related to urban economic development, environmental pollution, and well-being levels [13]. Urban energy utilization is reflected in various related subsystems of urban circular operations and developments [14,15]. Furthermore, urban energy utilization has affected the well-being level to a certain extent, and it is imperative to improve urban energy performance. Therefore, studying the concept and measurement results of urban energy performance from the perspective of system correlation is necessary in order to find ways to improve urban energy performance and determine future research directions.

This study was carried out in order to clarify the evolution characteristics and direction of the urban energy performance concept, reveal the research dimensions, determine the performance results and differences, and clarify the reference benchmark. The main contributions of this study are reflected in the following aspects: firstly, guided by the system view, this paper explains the concept evolution of urban energy performance and proposes the main dimensions of urban energy performance based on an in-depth analysis of the association network of the urban energy utilization system and existing research results. Secondly, combined with the basic characteristics of each dimension, this paper constructs the measurement indicator system and measurement model of urban energy performance in different dimensions. Thirdly, through the comparison and analysis of the energy performance measurement results of China’s prefecture level cities in different dimensions, the specific levels and overall differences in energy performance are clarified, and the benchmark cities for improving energy performance in a single dimension and multiple dimensions in China’s provinces are determined in order to provide a reference for cities around the world on how to improve their energy performance. Finally, the “chain” framework evolution direction of the concept and measurement of urban energy performance is proposed. It highlights the intermediate roles of economic output and environmental pollution. Thus, it considers the energy performance of the economic and environmental dimensions on the premise of ultimately improving the well-being level.

## 2. Conceptual Evolution of Urban Energy Performance from the Perspective of System Correlation

**Figure 1:**
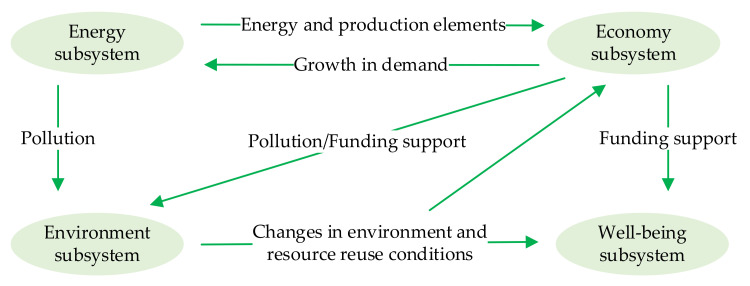
Multi-system network relationships of urban energy utilization impacts.

Since the introduction of the growth limit theory, energy, the economy, and the environment have attracted increasing attention and have gradually developed into a related binary theoretical system. Since the 1980s, the concept of sustainable development has been continuously improved. Furthermore, research focused on the three aforementioned aspects, such that the “energy-economy-environment” framework (i.e., “3E” system research framework) was established [16]. On this basis, scholars developed the “economy-society-ecology” composite system and integrated the social subsystem into the “3E” framework [17], which had something in common with the eco-city theory, which emphasized the reciprocal symbiosis of people, nature and city [18], transformed the focuses from land, transportation and species diversity to economy, law, social equity, lifestyle and public awareness, and extended to the well-being subsystem. Therefore, combined with the above analysis, it is concluded that urban energy utilization would have an impact on multiple urban subsystems, as shown in Figure 1.

The energy subsystem provides important energy and production elements for the economy subsystem and supports its development [19,20,21]. However, when energy use forms economic outputs, the pollution produced has an adverse impact on the environment subsystem [22]. The development of the economy subsystem increases energy demand, but it also provides effective support for environmental governance [23] and the improvement of social well-being levels. Environmental governance and environmental pollution would lead to both positive and negative changes in environment [24] and resource reuse conditions, which would affect the economy subsystem [25] and the well-being subsystem [26].

Following the above system correlation diagram, this paper analyzes the conceptual evolution of urban energy performance based on the current research results of urban energy performance. The concept of energy performance originated from research on the concept of energy efficiency. From the late 1970s, energy efficiency was a topical research issue. Energy efficiency was generally expressed by the ratio of energy service output to energy input [27]. Some scholars believe that energy efficiency is a relative concept, which must be quantified by certain indicators in order to change energy efficiency. Furthermore, the initial concept of energy performance was defined as using less energy to produce the same amount of services or useful outputs [28,29]. Thus, the determination of “output” has become a key element in defining energy performance.

After the industrial revolution, the production system based on fossil energy created great wealth. Energy became an important production element in human production activities and could be used to produce products by combining with capital, labor, and other production elements [29]. Therefore, the measurement of energy performance initially focused on economic outputs. However, due to the change of development environment and the fact that most traditional energy is nonrenewable, the development mode relying on element input is difficult to achieve sustainable growth. Thus, “Schumpeter growth” with structural transformation and technological innovation as the main driving forces was chosen. It could be concluded that energy economic performance is the initial category of the concept of energy performance. The purpose of energy economic performance is mainly to maximize economic output through the optimal combination of different elements such as capital input, labor input, and energy input [30], and it achieves this purpose by means of structural transformation, technological innovation, the optimization of economic development mode, and changes in other elements affecting economic growth. Accordingly, when economists analyzed energy performance, energy input was regarded as one of a variety of inputs [31], and the role of input element substitution in realizing energy performance was considered [32]. Therefore, the relative performance is mainly analyzed according to the distance between the samples and the production frontier. The definition and measurement of energy performance reveal the relationships between the energy subsystem and the economic subsystem.

In the above definitions, production elements such as capital, labor, and energy are considered as constraints when defining urban energy performance. However, the pollutants generated by energy use are ignored, and thus the undesirable output and environmental impact caused by energy use are also ignored [33,34,35]. The inefficiency of production technology leads to the generation of pollutants, and environmental performance is determined by pollutant emission reduction and its cost. Therefore, research on how pollutant emission reduction affected output began, and a model based on directional distance function was proposed [36]. The model effectively fitted the restrictive role of environmental impact in production, and made it possible to study the real effect of environmental impact [37,38]. To date, the research on energy performance has focused on multiple-inputs and multiple-outputs. The environmental production function and environmental directional distance function are integrated into the exploration of environmental performance in the production process, in order to reduce input, expand output, reduce pollutants, and realize the optimal combination of elements under this condition [39]. The definition and measurement of energy performance are extended from the impact on the economic subsystem to the impact on the environmental subsystem.

The traditional well-being economics of utility theory pointed out that consumer surplus could be used to reflect the level of social well-being, thus, the scheme that can increase consumer surplus may become Pareto optimal, which reflects the significance and idea of improving energy performance. Ecological environment is a typical public good with the characteristics of non-exclusivity, externality, non-competitiveness, and unclear property rights. Energy resources also belong to public goods in terms of use [40]. Thus, the consumption of natural capital may be higher than the best social consumption value [41]. Existing studies have focused on the impact of environmental pollution on economy, such as ecological compensation [42], green GDP estimation [43], and other related studies. These studies mainly focus on the resource allocation role of price, but human health and well-being belong to the component of “function”, which is different not only from the concept of commodity, but also from the concept of utility. Therefore, urban energy performance evaluation should not only consider the monetary factors in the traditional well-being economic analysis, but also integrate the well-being level into the research, in order to ensure the urban energy performance indicators have stronger explanatory power. Therefore, the definition and measurement of energy performance are related to the well-being subsystem.

Based on different social backgrounds and theoretical foundations, scholars have proposed the corresponding concept of urban energy performance, but there are also the following research gaps: firstly, although with the increase in research depth, the reasonable energy performance concepts under different social backgrounds and theoretical basis have been put forward, the research dimensions of urban energy performance have not been determined. Secondly, because the research dimension has not been clearly determined, the actual measurement results of urban energy performance under different dimensions are unknown. It is difficult to find a benchmark city and focus to improve urban energy performance. Thirdly, based on the above review, it has been found that there are differences and connections in the concept of urban energy performance under different social backgrounds and theoretical bases. Therefore, it is necessary to further clarify the specific relationships and propose a new conceptual framework of urban energy performance. The solution of these research gaps makes up for the existing research results, thus it is novel. It not only develops effective dimensions and conceptual frameworks for future research on urban energy performance, but also objectively, accurately, and comprehensively reflects the performance results and promotion orientations of different cities and different dimensions, thus, it is of great significance.

**Figure 2:**
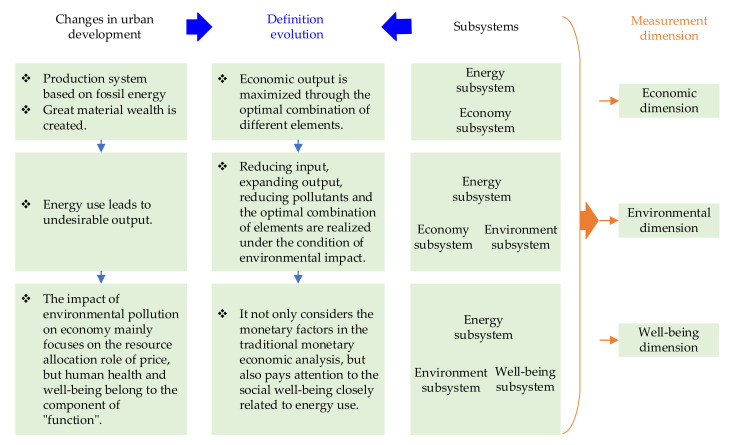
Concept evolution and dimension of urban energy performance.

Therefore, based on the above analysis, the concept evolution of urban energy performance is summarized, and then the main dimensions of urban energy performance measurement are determined, as shown in Figure 2.

## 3. Measurement Indicators and Models of Urban Energy Performance in Different Dimensions

The core research questions are to distinguish the conceptual evolution of urban energy performance from the perspective of system correlation and compare urban energy performance from multiple dimensions. Therefore, based on the above review and summary of the concept of urban energy performance, the measurement dimension of urban energy performance mainly includes three aspects: the economic dimension, the environmental dimension, and the well-being dimension. Furthermore, this section will determine the measurement indicators of urban energy performance under different dimensions in combination with literature review results, and then construct the measurement models of urban energy performance in different dimensions based on the corresponding model using the Data Envelopment Analysis method.

The urban energy performance measurement in the economic dimension tends to reflect the maximum economic output achieved under a certain energy input and other inputs. Moreover, the measurement in the existing research is mainly based on the energy input and other inputs required in production, as well as the output value that can be generated. In terms of urban energy performance measurement, the measurement indicators in the economic dimension mainly include energy consumption [44], fixed capital investment [45], labor input [46], and economic output [27]. This is a measurement indicator system based on the basic production function relationship and the total factor analysis framework.

The measurement of urban energy performance in the environmental dimension is also based on the total factor analysis framework, but it aims to achieve the optimal output. That is, it not only maximizes economic output, but also minimizes environmental impact. For the indicators, the input indicators still take energy consumption, fixed capital input and labor input as the input indicators. However, the output indicators include not only gross domestic product, but also carbon dioxide emissions [47], sulfur dioxide emissions [36,48], nitrogen oxides, chemical oxygen demand, solid waste generation [49], etc. Therefore, the measurement of urban energy performance in the environmental dimension not only reflects the expected output of urban energy utilization, but also covers the unexpected output.

The core meaning of the concept of urban energy performance in the well-being dimension is reflected in that urban development aims to improve the well-being level by a certain energy supply [50,51]. Therefore, scholars also choose energy consumption as the input indicator to measure energy performance from its fundamental meaning. However, some scholars believe that in order to improve the well-being level, cities would inevitably produce pollution emissions in the process of energy consumption, which is also the cost that cities need to pay when improving the well-being level [52]. Therefore, some scholars have added environmental pollution indicators (or environmental pressure indicators) to the input indicators, which are mostly expressed by urban “three wastes” (wastewater, waste gas, and solid waste) [52] or carbon emission [53,54]. In addition, in order to better reflect the capital utility invested to maintain ecological balance, Xiao and Zhang included environmental capital indicators, service-oriented capital indicators, and resource-based capital indicators in their research [55]. In terms of selecting output indicators, more scholars choose indicators based on the comprehensive consideration of medical and health level [56], educational level, and economic development level [57] in view of the integrity and availability of objective well-being indicator data [51,58]. Based on the above analysis, the input indicators selected in this study include energy consumption, environmental pollution, and environmental capital investment.

**Figure 3:**
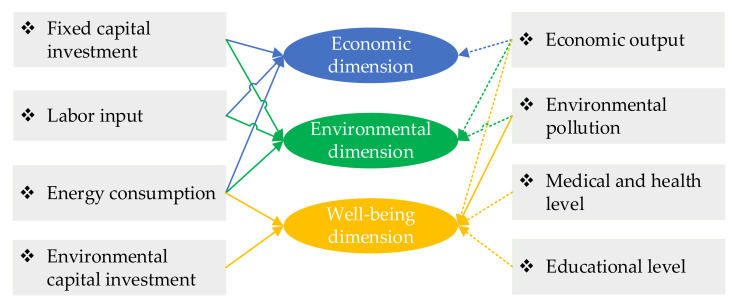
The measurement indicators of urban energy performance in different dimensions. Note: the blue, green and yellow arrow lines represent the measurement indicators of economic, environmental, and well-being dimensions, respectively. The solid lines represent the input indicators, and the dotted lines represent the output indicators.

The summary of urban energy performance measurement indicators in different dimensions is shown in Figure 3.

From the above determined measurement indicators, it can be demonstrated that the measurement model of urban energy performance in different dimensions needs to meet different measurement requirements. In the environmental dimension and well-being dimensions particularly, it is necessary that the model can measure multi-output indicators and the unexpected output indicator. As an effective performance measurement method, Data Envelopment Analysis has been continuously improved and developed from many aspects since it was proposed in 1978, and it has formed an important methodology system that can effectively deal with a variety of outputs, a variety of returns to scale, relaxation measurements, super efficiency problems, mixed efficiency, non-discretionary, uncontrollable situations, classified demand, unexpected output, dynamic panel data, multi-stage situation, and other problems [59]. Therefore, this study will construct urban energy performance measurement models in different dimensions based on the Data Envelopment Analysis method and the corresponding measurement indicators.

The measurement of urban energy performance in the economic dimension aims to reflect the effect of urban energy utilization based on the basic production function. The return to scale of urban energy utilization is variable and is not in the most large-scale production state. Therefore, the construction of the urban energy performance measurement model in the economic dimension is based on the variable returns to scale radial Data Envelopment Analysis model [60].

It is supposed that there are *n* cities in the measurement. Each city has *m* inputs and *q* outputs. The *i*th input of the *j*th (*j* = 1, 2, …, *n*) city is expressed as *x_i__j_* (*i* = 1, 2, …, *m*), and the *r*th output of the *j*th city is expressed as *y_r__j_* (*r* = 1, 2, …, *q*). The city to be measured is set as the *k*th city currently. Therefore, the measurement model of urban energy performance in the economic dimension could be set as [61]:

(1) $$\begin{array}{l} \min\;\theta \\ s.t.\;\overset{n}{\underset{j=1}{\sum }}\lambda_{j}x_{ij}\leq \theta x_{ik}\\ \;\;\;\;\;\;\;\;\overset{n}{\underset{j=1}{\sum }}\lambda_{j}y_{rj}\geq y_{rk}\\ \;\;\;\;\;\;\;\;\overset{n}{\underset{j=1}{\sum }}\lambda_{j}=1\\ \;\;\;\;\;\;\;\;\lambda \geq 0\\ \;\;\;\;\;\;\;\;i=1,\;2,\;\ldots ,\;m;\;r=1,\;2,\;\ldots ,\;q;\;j=1,\;2,\;\ldots ,\;n \end{array}$$

In Equation (1), *λ* represents the linear combination coefficient of the decision-making unit. $\theta^{*}$ is the optimal solution of the model and it represents the efficiency value. Its value range is (0, 1]. The smaller $\theta^{*}$ is, the larger the reduction range is and the lower the efficiency is. When $\theta^{*}$ = 1, the decision-making unit is located on the optimal frontier. Under the condition of not reducing output, there is no room for the proportional decline of the decision-making unit’s inputs, and the decision-making unit is in a state of technical efficiency. If $\theta^{*}$ < 1, it is indicated that the decision-making unit is in the state of technical inefficiency. Under the condition of not reducing output, the proportion that each input of the decision-making unit can decrease in equal proportion is (1−$\theta^{*}$).

Based on the above analysis, the measurement of urban energy performance in the environmental dimension needs to reflect the unexpected output. Therefore, the measurement model is constructed based on the directional distance function [62]. In the environmental dimension, the input is set as *H*, the expected output is set as *O*, and the unexpected output is set as *B*, so the corresponding expected output vector is set as $g_{o}$ and the unexpected output vector is $g_{b}$. The city to be measured is set as the *l*th city currently, thus the model is expressed as Equation (2):

(2) $$\begin{array}{l} \max\;\beta \\ s.t.H\delta +\beta g_{h}\leq h_{l}\\ \;\;\;\;\;\;\;\;O\delta -\beta g_{o}\leq o_{l}\\ \;\;\;\;\;\;\;\;B\delta +\beta g_{b}\leq b_{l}\\ \;\;\;\;\;\;\;\;\sum \delta =1\\ \;\;\;\;\;\;\;\;\delta \geq 0,\;g_{h}\geq 0,\;g_{o}\geq 0,\;g_{b}\geq 0 \end{array}$$

In Equation (2), −$g_{h}$, $g_{o}$, −$g_{b}$ are the direction vectors of input, the expected output, and the unexpected output, respectively, which means that the evaluated city aims to reduce input, increase expected output, and reduce unexpected output. *β* reflects the analysis results of directional distance function.

The urban energy performance measurement in the well-being dimension aims to reflect the impact of energy use on the well-being level. The above conceptual analysis and indicator determination also reflects the derived input indicators, namely environmental capital investment and environmental pollution. In this scenario, the return to scale of energy utilization in the well-being dimension is also variable. Therefore, the construction of the urban energy performance measurement model in the well-being dimension is still based on the variable returns to scale radial Data Envelopment Analysis model. It is supposed that there are *a* cities in the measurement. Each city has *s* inputs and *t* outputs. The *d*th input of the *c*th (*c* = 1, 2, …, *a*) city is expressed as *u_dc_* (*d* = 1, 2, …, *s*), and the *e*th output of the *c*th city is expressed as *v_ec_* (*e* = 1, 2, …, *t*). The city to be measured is set as the *f*th city currently. Therefore, the measurement model of urban energy performance in the well-being dimension could be set as [61]:

(3) $$\begin{array}{l} \min\;\mu \\ s.t.\;\overset{a}{\underset{c=1}{\sum }}\varphi_{c}u_{dc}\leq \mu u_{df}\\ \;\;\;\;\;\;\;\;\overset{a}{\underset{c=1}{\sum }}\varphi_{c}v_{ec}\geq v_{ef}\\ \;\;\;\;\;\;\;\;\overset{a}{\underset{c=1}{\sum }}\varphi_{c}=1\\ \;\;\;\;\;\;\;\;\varphi \geq 0\\ \;\;\;\;\;\;\;\;d=1,\;2,\;\ldots ,\;s;\;e=1,\;2,\;\ldots ,\;t;\;c=1,\;2,\;\ldots ,\;a \end{array}$$

In Equation (3), *μ* represents the linear combination coefficient of the decision-making unit. $\mu^{*}$ is the optimal solution of the model and it represents the efficiency value. Its value range is (0, 1]. The smaller $\mu^{*}$ is, the larger the reduction range is and the lower the efficiency is. When $\mu^{*}$ = 1, the decision-making unit is located on the optimal frontier. If $\mu^{*}$ < 1, it is indicated that the decision-making unit is in the state of technical inefficiency. Under the condition of not reducing output, the proportion that each input of the decision-making unit can decrease in equal proportion is (1 − $\mu^{*}$).

The measurement period of urban energy performance in this study is 2018. The performance differences between different cities in the same dimension are reflected by standard deviation, and the urban energy performance differences between different dimensions are obtained by comparing the measurement results, minimum values, mean values, and standard deviations of cities.

## 4. Multi-Dimensional Measurement Results and Discussion of Energy Performance of Prefecture Level Cities in China

### 4.1. The Range of Study Sample and the Variables of Indicators

Since the reform and opening up, China’s urbanization level has been continuously improved, from 17.92% in 1978 to 59.58% in 2018. The improvement in urbanization level has not only increased China’s energy consumption, but has also driven economic and social development and formed an impact on the environment, which fully reflects the correlated role of energy utilization in each subsystem and the positive and negative utilities caused by energy utilization in the process of urban development. Secondly, China’s primary energy consumption reached 24.7% of the global primary energy consumption in 2018 [52]. The emergence of ecological and environmental problems made people recognize the importance of improving energy performance [63], and also demonstrated the urgency of improving urban energy performance in China. Thirdly, China has a vast territory and a large number of cities which demonstrate differences in energy, economy, environment, society, and other aspects. The research on China’s prefecture level cities can fully reflect the multi-dimensional energy performance level of cities under different development situations and determine the energy performance level for different cities in the world, which provides an important reference for formulating energy performance improvement policies. Therefore, this study takes 142 prefecture level cities in China as samples to measure and compare their multi-dimensional energy performance.

In view of the availability of a large sample of urban indicator data and the characteristics of urban energy utilization, the effective variables are selected for urban energy performance measurement indicators in these three dimensions. As the energy consumption of urban development mainly focuses on industrial energy consumption, the energy consumption indicator in this study is reflected by the energy consumption of industrial enterprises above designated size. The labor input is expressed by the total number of employees in urban units and urban private and individual employees. In cities, industrial sulfur dioxide and industrial nitrogen oxides are the main sources of pollution, thus, the environmental pollution is expressed as the sum of the two emissions. However, the life expectancy and the years of education of residents in many cities are difficult to obtain. The measurement of urban education level is characterized by the years of education supply. The calculation method is shown in Equation (4) [64]:

(4) $$W=\;\left(6\times p_{1}+9\times p_{2}+12\times p_{3}+16\times p_{4}\right)/\left(p_{1}+p_{2}+p_{3}+p_{4}\right)$$

In Equation (4), *W* represents the educational capacity that the city can provide, that is, the years of education supply. *p*_1_, *p*_2_, *p*_3_, and *p*_4_ represent the number of students in ordinary primary schools, ordinary middle schools, secondary vocational education school, junior colleges, and universities, respectively. At present, there is no official data on the total investment in urban pollution control, and the investment in environmental control of each city is closely related to the economic level and the degree of environmental pollution. Therefore, the setting of the total investment in pollution control of each city in this study is mainly based on this principle and the investment in pollution control of each province in China. The medical and health level of a city is expressed according to the medical conditions that the city can provide, that is, the number of practicing (assistant) doctors. All data were collected from China Urban Statistical Yearbook, China Environmental Statistical Yearbook, and various urban statistical yearbooks. Some missing data were supplemented by interpolation methods and trend analysis methods. There is a descriptive statistical analysis on the indicator data of 142 sample cities in Table 1. It can be seen that the 142 prefecture level cities in China have different numerical levels in each indicator, which further confirms that the large sample study of prefecture level cities in China can help more cities to determine the rationality, effectiveness, and reference of energy performance level measured based on the numerical levels of input and output indicators in different dimensions, and reflects the correctness of selecting prefecture level cities in China for empirical research.

### 4.2. The Measurement Results of Urban Energy Performance in the Economic Dimension

Based on the measurement indicators and models of urban energy performance in the economic dimension, the measurement results of energy performance of prefecture level cities in China in the economic dimension are obtained, as shown in Table 2.

According to the analysis of Table 2, 22 cities are in an effective state, namely Tianjin, Tangshan, Shuozhou, Dalian, Siping, Shanghai, Taizhou (in Jiangsu province), Weihai, Changsha, Zhangjiajie, Guangzhou, Foshan, Dongguan, Zhongshan, Guilin, Haikou, Sanya, Xianyang, Yulin, Jiayuguan, Zhangye, and Turpan. The other cities have room to improve energy performance in the economic dimension. Among the prefecture level cities, Shantou had the lowest energy performance, with a performance value of 0.3843. The average energy performance in the economic dimension is 0.7399, and the overall performance level is relatively high. The standard deviation is 0.1799, and the gap between cities is relatively small.

### 4.3. The Measurement Results of Urban Energy Performance in the Environmental Dimension

Similarly, the energy performance values of China’s prefecture level cities in the environmental dimension are obtained by using the measurement indicators and models in the third part, as shown in Table 3.

The measurement results of urban energy performance in the environmental dimension show that 28 cities are in an effective state, including Beijing, Tianjin, Tangshan, Cangzhou, Shuozhou, Dalian, Siping, Shanghai, Zhenjiang, Taizhou (in Jiangsu province), Qingdao, Weihai, Suizhou, Changsha, Zhangjiajie, Guangzhou, Foshan, Dongguan, Zhongshan, Guilin, Haikou, Sanya, Xianyang, Yulin, Jiayuguan, Zhangye, Karamay, and Turpan. Therefore, 114 cities need to improve their performance levels. Among these prefecture level cities, Shantou still had the lowest energy performance, and the lowest performance value is 0.3819. The average value of energy performance in the environmental dimension is 0.7596, which is generally at a relatively good performance level. The standard deviation is 0.1832.

### 4.4. The Measurement Results of Urban Energy Performance in the Well-Being Dimension

After analyzing the sample cities by using the measurement indicators and models of urban energy performance in the well-being dimension set in this study, Table 4 shows the measurement results of energy performance of prefecture level cities in China in the well-being dimension.

In the well-being dimension, 16 sample cities are in an effective state, including Beijing, Changchun, Shanghai, Nanjing, Nanchang, Zhoukou, Wuhan, Suizhou, Changsha, Xiangtan, Guangzhou, Haikou, Sanya, Chengdu, Xi’an, and Lanzhou. Cities that are not in an effective state still have room to improve their performance level. Unlike the first two dimensions, Jincheng had the lowest energy performance value in the well-being dimension. In addition, the average value of energy performance in the well-being dimension is 0.3605, and the overall performance level is low. The standard deviation is 0.3101, and the performance level gap between sample cities is relatively large.

### 4.5. Results Comparison of Urban Energy Performance in Different Dimensions and Discussion

After the descriptive statistical analysis of the measurement results of urban energy performance in different dimensions, it was found that the average energy performance of prefecture level cities in China in the economic dimension is close to that in the environmental dimension, but there is still a gap between them, with difference of 0.0197. The performance gap between cities in these two dimensions is also relatively similar. The reason is that the energy performance measurement indicator difference between the economic dimension and the environmental dimension is relatively small, and the environmental pollution is further considered in the urban energy performance measurement in the environmental dimension. In addition, through further analysis, it can also be demonstrated that the awareness of energy conservation and environmental protection is improved, and the energy conservation and emission reduction technology is developed with the economic and social development. Therefore, the negative impact of environmental pollution on urban energy performance had also improved to a certain extent in 2018. However, there is a large gap between the measurement results in the economic dimension and the environmental dimension and those in the well-being dimension. The mean value in the well-being dimension is nearly 50% lower than that in the economic dimension. From the well-being dimension, the energy performance of China’s prefecture level cities still has great room for improvement. In addition, the performance gap between cities in the well-being dimension is also significantly higher than that in the economic dimension and the environmental dimension. The increase in energy performance difference in the well-being dimension is related to the urban economic development difference, infrastructure construction, pollution control, social security, and so on. How to further narrow the gap between cities on the premise of improving the overall energy performance level of prefecture level cities in China in the well-being dimension is a key area which is worthy of further study. 

On the other hand, for the number of cities that have effective energy performance in different dimensions, there is a small difference between the environmental dimension and the economic dimension, and there is a large gap between the well-being dimension and the first two dimensions. Therefore, from the perspective of China’s overall improvement of energy performance, it is necessary to pay further attention to the well-being dimension in the future. Furthermore, through the comparison of effective energy performance cities in each dimension, it is demonstrated that the cities that reach the performance frontier in the economic dimension also reach the performance frontier in the environmental dimension. Compared with the cities in the economic dimension, there are six cities added into the environmental dimension, namely Beijing, Cangzhou, Zhenjiang, Qingdao, Suizhou, and Karamay. It further shows that the six cities not only improve economic output, but also control the emission of environmental pollutants. Compared with the cities with effective energy performance in the economic dimension and the environmental dimension, the cities with effective energy performance in the well-being dimension differ greatly. It is found that Beijing, Changchun, Nanjing, Zhoukou, Wuhan, Suizhou, Xiangtan, Chengdu, Xi’an, and Lanzhou have also reached the effective frontier compared with the cities with effective energy performance in the economic dimension. Overall, Shanghai, Guangzhou, Changsha, Haikou, and Sanya have reached the effective frontier in terms of energy performance in the three dimensions.

According to the ranking of each city in different dimensions, the performance level of each city could be further analyzed in multiple dimensions and the differences between different dimensions could be further considered. Although Shanghai, Guangzhou, Changsha, Haikou, and Sanya have reached the performance frontier in the three dimensions, the energy performance of Beijing, Tianjin, Dalian, Changchun, Siping, Taizhou (in Jiangsu province), Qingdao, Zhoukou, Wuhan, Xiangtan, Zhangjiajie, Foshan, Dongguan, Zhongshan, Guilin, and Chengdu have also reached a higher performance level compared with other cities in the three dimensions. The ranking of these cities is relatively high in each dimension, and these cities have reached the performance frontier in at least one dimension. Although Tangshan, Cangzhou, Shuozhou, Zhenjiang, Weihai, Xianyang, Yulin, Jiayuguan, Zhangye, Karamay, and Turpan have reached the performance frontier in one of the three dimensions, the performance level of these cities in the well-being dimension is relatively low, which is in great contrast to the performance level in the economic dimension and the environmental dimension. Therefore, these cities need to focus on improving energy performance in the well-being dimension. Correspondingly, the performance level of Nanjing, Nanchang, Suizhou, Xi’an, and Lanzhou in the well-being dimension has reached the effective frontier, while the performance level in the economic dimension or the environmental dimension is relatively low.

To achieve the improvement goals of different cities, it is necessary to further clarify the target cities that can be used for reference. In view of the similarity of geographical location and economic and social development level, this study summarizes the benchmark city of each province, as shown in Table 5.

In the economic dimension, the benchmark cities of Hebei, Shanxi, Liaoning, Jilin, Jiangsu, Anhui, Fujian, Shandong, Henan, Guangxi, Sichuan, Shaanxi, Gansu, Ningxia and Xinjiang, are non-provincial capital cities. In the environmental dimension, the benchmark cities of Hebei, Shanxi, Liaoning, Jilin, Jiangsu, Fujian, Shandong, Henan, Hubei, Guangxi, Sichuan, Shaanxi, Gansu, Ningxia, and Xinjiang are non-provincial capital cities. Compared with the economic dimension, Anhui province is not included, while Hubei province is included. Therefore, in the economic and environmental dimension, 15 performance benchmark cities of the provinces where the sample cities are located are non-provincial capital cities, respectively. However, in the well-being dimension, the benchmark cities in Hebei, Liaoning, Fujian, Henan, and Ningxia are non-provincial capital cities. Considering the multiple dimensions, the benchmark cities of Shanxi, Jilin, Jiangsu, Shandong, Guangxi, Gansu, Xinjiang, and the above five provinces are also non-provincial capital cities. It can be seen that the benchmark cities of most provinces are provincial capital cities in the well-being dimension. The analysis and determination of benchmark cities will provide some reference for improving the energy performance of each province.

**Figure 4:**
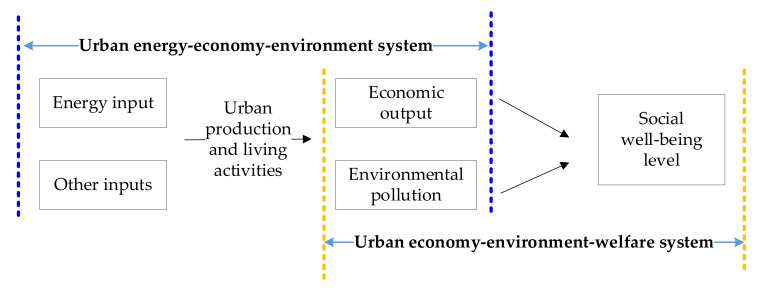
The evolution direction of the conceptual framework of urban energy performance.

Based on the analysis of the energy-economy-environment-welfare system in a city and the review of the concept of urban energy performance from the perspective of system correlation, this paper further proposes the different dimensions of urban energy performance measurement and the measurement indicators in each dimension. From the analysis results of the measurement indicators, it is concluded that the measurement indicators in the environmental dimension include the unexpected output indicator. Therefore, the bad output caused by energy use in the city is comprehensively considered. In addition, the input of environmental pollution control comes from the urban economic output, and the urban environmental pollution will also have an important impact on the urban well-being level. This further reflects the continued relationship between the environmental dimension and the well-being dimension, provides an important direction for the research on the concept and indicators of urban energy performance, and also lays a certain theoretical foundation for the research on urban energy performance. At the same time, based on the analysis of the multi-dimensional measurement results of China’s prefecture level cities, it is concluded that the measurement results of urban energy performance in the economic and environmental dimensions are relatively similar, but there is a large gap between these two dimensions and the well-being dimension, which further verifies the extended relationship between the environmental dimension and the economic dimension reflected in the conceptual analysis and shows the significant impact that environmental pollution control investment and environmental pollution will make on energy performance results [52,65,66]. In order to further reflect the utility of energy in urban production, the adverse utility of environmental pollution and the boosting effect of economy on environmental governance and well-being level improvement in the basic production theory, it gradually evolves into the conceptual framework of urban energy performance shown in Figure 4. It presents a “chain” basic framework assumption. In the measurement of urban energy performance, it will gradually be transformed into the ultimate orientation of improving the well-being level, and the economic output and environmental impact caused by energy use will be contained in the comprehensive correlation system of urban energy use.

## 5. Conclusions

Based on the conceptual analysis of urban energy performance guided by systematic research and previous literature, this study summarizes the evolution trend of the concept of urban energy performance, and proposes the main dimensions of urban energy performance measurement. On this basis, the measurement indicators of urban energy performance in different dimensions are determined, and the measurement model of each dimension is constructed based on the Data Envelopment Analysis method. In addition, this paper empirically analyses 142 prefecture level cities in China. The conclusions are listed as follows: 

Firstly, the development of system theory and the deepening of the derivative effect and ultimate purpose of urban energy utilization make the research on urban energy performance into the related system of energy-economy-environment-welfare, which further highlights the multi-dimensional roles of urban energy consumption. Taking this as a guide, this paper analyzes the concept of urban energy performance, reveals the basic conceptual framework of energy performance characterized by “input-output”, presents the research trend of measuring economic wealth, environmental impact and well-being level as “output”, and puts forward the main dimensions of urban energy performance research, including the economic dimension, the environmental dimension and the well-being dimension.

Secondly, based on the literature review, this paper also puts forward the input indicators and output indicators of urban energy performance measurement in different dimensions. The input indicators in the economic dimension include energy consumption, labor input, and fixed capital input, and the output indicators include economic output. The measurement indicators of urban energy performance in the environmental dimension add environmental pollution on the basis of the output indicators in the economic dimension. The input indicators in the well-being dimension include energy consumption, environmental capital investment, and environmental pollution, while the output indicators include economic output, medical and health level, and educational level. In view of the characteristics of measurement indicators in different dimensions, the measurement models of urban energy performance in different dimensions are constructed based on the corresponding models in the Data Envelopment Analysis method, which not only reduces the result error caused by the unreasonable setting of production function, but also effectively deals with the unexpected output.

Thirdly, the research carried out on 142 prefecture level cities in China fully considers the energy performance levels of cities with different development levels. Through multi sample research, it further improves the effectiveness and rationality of the empirical research and provides an important reference for different cities in the world to improve their energy performance of different dimensions. The empirical results show that the energy performance level of prefecture level cities in China in the economic dimension and the environmental dimension is relatively close, but there are some differences between the urban energy performance level in the well-being dimension and the first two dimensions. In addition, the performance difference between cities in the well-being dimension is also greater than the first two dimensions. In the three dimensions, there are 22, 28 and 16 cities that have reached the effective energy performance, respectively. Shanghai, Guangzhou, Changsha, Haikou, and Sanya have reached the effective frontier in the three dimensions. Among these cities, Beijing, Tianjin, Dalian, Changchun, Siping, Taizhou (in Jiangsu province), Qingdao, Zhoukou, Wuhan, Xiangtan, Zhangjiajie, Foshan, Dongguan, Zhongshan, Guilin, and Chengdu rank relatively high in all dimensions. However, the performance level of Tangshan, Cangzhou, Shuozhou, Zhenjiang, Weihai, Xianyang, Yulin, Jiayuguan, Zhangye, Karamay, and Turpan in the well-being dimension is relatively low, which forms a large contrast with the performance level in the economic dimension and the environmental dimension. The performance level of Nanjing, Nanchang, Suizhou, Xi’an, and Lanzhou in the economic dimension or environmental dimension is relatively poor compared with that in the well-being dimension. Based on the detailed analysis of each dimension, this paper clarifies the benchmark cities in various provinces of China for the purpose of improving their performance. In the economic and the environmental dimension, 15 performance benchmark cities in the provinces where the sample cities are located are non-provincial capital cities, respectively. In the well-being dimension, the benchmark cities in Hebei, Liaoning, Fujian, Henan, and Ningxia are non-provincial capital cities. From the multiple dimensions, the benchmark cities in 12 provinces are non-provincial capital cities. The determination of benchmark cities in each province can provide an effective reference for each region to improve urban energy performance.

The limitations of this study mainly focus on the difficulty of data collection. Based on the scientific analysis of the concept of urban energy performance, this paper puts forward the measurement dimensions of urban energy performance and the measurement indicators under different dimensions. Although the 142 sample cities in China selected in the empirical study have different levels of energy, economy, environment, and well-being and the performance level of global cities in different dimensions could be effectively reflected based on these cities to some extent, there is still a lack of comprehensive analysis on the global cities, which is mainly due to the difficulty of data collection. It is difficult to obtain all the data of various indicators in some cities under the three dimensions. Therefore, future research should focus on the measurement and comparison of multi-dimensional urban energy performance for global cities to comprehensively reveal the global urban energy performance level. Secondly, improving urban energy performance in different dimensions is the most important research direction after scientifically determining the performance level. Therefore, future research should also focus on urban energy performance improvement policies and measures, including the evaluation of policy effectiveness, the quantitative analysis of policy methods, the design of improvement paths, and other aspects.

## 6. Policy Recommendations

Based on the above conclusions, this study puts forward the following policy recommendations:

Firstly, multi-dimensional urban energy performance measurement is closely related to the energy-economy-environment-welfare system of urban development, and there is a need for continuous observation and analysis. There are a large number of cities in the world. In order to effectively record, measure, and monitor the data and analysis results related to urban energy performance, it is necessary to construct a global urban energy performance database and to further realize global energy performance data management by using advanced computer technology and internet technology.

Secondly, based on the concept deduction and urban subsystem analysis, or the energy performance measurement of different dimensions of China’s prefecture level cities, the urban energy performance in the well-being dimension should be given careful attention in order to be improved in the future. Therefore, on the premise of the rational use of energy to create economic output, cities should also reduce their emission of environmental pollutants, to further reduce the negative impacts on urban environmental governance input and urban residents’ health. Furthermore, cities should ensure the investment and improvement of human development, such as residents’ education.

Thirdly, based on the energy performance measurement results of each dimension, it is concluded that the energy performance of China’s prefecture level cities still has great potential to improve in each dimension, particularly in the well-being dimension. The determination of benchmark cities in this study can help cities in similar areas to determine goals. On the basis of comprehensively considering the similarity of various indicators, it is necessary to seek advanced experience to improve urban energy performance in all dimensions from regional, economic, environmental, social and other aspects more pertinently and effectively.

## Figures and Tables

**Table 1:** The descriptive statistical analysis of indicator data.

Indicator	Unit	Minimum	Maximum	Mean	Standard Deviation
Energy consumption	10,000 tons of standard coal	11.87	18,102.33	2041.73	2653.59
Labor input	Person	138,105.00	15,696,019.00	1,989,929.00	2,420,450.00
Fixed capital investment	Million Yuan	119.20	18,661.41	2958.72	2543.64
Economic output	Ten thousand Yuan	2,996,200.00	326,798,700.00	44,685,392.00	52,717,490.00
Environmental pollution	Ton	412.00	249,071.00	37,880.48	39,823.75
Environmental capital investment	Ten thousand Yuan	440.71	190,731.81	27,999.97	31,410.93
Medical and health level	Person	993.00	109,376.00	15,814.42	15,111.48
Education level	Year	7.46	11.76	8.76	1.11

**Table 2:** The measurement results of energy performance of prefecture level cities in China in the economic dimension.

City	Performance	City	Performance	City	Performance
Beijing	0.9952	Longyan	0.8362	Zhuhai	0.7241
Tianjin	1.0000	Ningde	0.6474	Shantou	0.3843
Tangshan	1.0000	Nanchang	0.6634	Foshan	1.0000
Handan	0.4883	Jingdezhen	0.5420	Maoming	0.7352
Baoding	0.6988	Jiujiang	0.6147	Zhaoqing	0.4492
Cangzhou	0.7934	Ganzhou	0.4643	Shanwei	0.5271
Taiyuan	0.6504	Shangrao	0.5841	Dongguan	1.0000
Yangquan	0.9334	Jinan	0.6936	Zhongshan	1.0000
Changzhi	0.5866	Qingdao	0.9620	Jieyang	0.8280
Jincheng	0.7376	Zaozhuang	0.6863	Yunfu	0.6764
Shuozhou	1.0000	Yantai	0.9976	Nanning	0.5456
Jinzhong	0.5200	Weifang	0.7912	Liuzhou	0.5520
Xinzhou	0.9677	Weihai	1.0000	Guilin	1.0000
Hohhot	0.7383	Rizhao	0.8191	Fangchenggang	0.8655
Dalian	1.0000	Linyi	0.4770	Haikou	1.0000
Changchun	0.7893	Dezhou	0.8379	Sanya	1.0000
Siping	1.0000	Binzhou	0.5409	Chongqing	0.7198
Harbin	0.9292	Zhengzhou	0.7933	Chengdu	0.8506
Shanghai	1.0000	Luoyang	0.6096	Zigong	0.9024
Nanjing	0.7063	Pingdingshan	0.5014	Luzhou	0.5563
Wuxi	0.9395	Anyang	0.4886	Deyang	0.9686
Xuzhou	0.7498	Xinxiang	0.5141	Guangyuan	0.5952
Suzhou	0.9169	Puyang	0.5439	Suining	0.7210
Nantong	0.7495	Sanmenxia	0.7258	Neijiang	0.9033
Lianyungang	0.7166	Nanyang	0.6128	Leshan	0.7260
Huai’an	0.7018	Shangqiu	0.5447	Guiyang	0.7262
Zhenjiang	0.9858	Xinyang	0.6210	Liupanshui	0.6984
Taizhou (in Jiangsu province)	1.0000	Zhoukou	0.9780	Bijie	0.5826
Suqian	0.9322	Wuhan	0.8874	Kunming	0.4679
Hangzhou	0.9355	Huangshi	0.4241	Xi’an	0.6929
Ningbo	0.6082	Shiyan	0.5512	Baoji	0.6891
Wenzhou	0.5971	Yichang	0.7037	Xianyang	1.0000
Shaoxing	0.7270	Xiangyang	0.8009	Weinan	0.5876
Jinhua	0.5588	Jingmen	0.5513	Yan’an	0.6431
Taizhou (in Zhejiang province)	0.7415	Suizhou	0.6085	Hanzhong	0.4882
Hefei	0.6893	Changsha	1.0000	Yulin	1.0000
Wuhu	0.6829	Zhuzhou	0.6900	Ankang	0.6999
Huainan	0.5241	Xiangtan	0.9192	Shangluo	0.5693
Bozhou	0.4784	Shaoyang	0.4815	Lanzhou	0.5247
Chizhou	0.6979	Changde	0.6805	Jiayuguan	1.0000
Xuancheng	0.5629	Zhangjiajie	1.0000	Zhangye	1.0000
Fuzhou	0.6558	Yiyang	0.9264	Yinchuan	0.5326
Xiamen	0.6723	Chenzhou	0.6192	Shizuishan	0.8587
Putian	0.4777	Yongzhou	0.5855	Urumqi	0.5936
Sanming	0.6734	Huaihua	0.6570	Karamay	0.9855
Quanzhou	0.7297	Guangzhou	1.0000	Turpan	1.0000
Zhangzhou	0.8130	Shaoguan	0.6634	Hami	0.9850
Nanping	0.6183				

**Table 3:** The measurement results of energy performance of prefecture level cities in China in the environmental dimension.

City	Performance	City	Performance	City	Performance
Beijing	1.0000	Longyan	0.8313	Zhuhai	0.7665
Tianjin	1.0000	Ningde	0.6442	Shantou	0.3819
Tangshan	1.0000	Nanchang	0.6638	Foshan	1.0000
Handan	0.5803	Jingdezhen	0.5387	Maoming	0.9919
Baoding	0.6992	Jiujiang	0.6148	Zhaoqing	0.4242
Cangzhou	1.0000	Ganzhou	0.4655	Shanwei	0.5842
Taiyuan	0.6408	Shangrao	0.5851	Dongguan	1.0000
Yangquan	0.9216	Jinan	0.6935	Zhongshan	1.0000
Changzhi	0.5856	Qingdao	1.0000	Jieyang	0.8596
Jincheng	0.7568	Zaozhuang	0.7604	Yunfu	0.6651
Shuozhou	1.0000	Yantai	0.9977	Nanning	0.5461
Jinzhong	0.5303	Weifang	0.9160	Liuzhou	0.5519
Xinzhou	0.9839	Weihai	1.0000	Guilin	1.0000
Hohhot	0.7294	Rizhao	0.8932	Fangchenggang	0.8428
Dalian	1.0000	Linyi	0.4755	Haikou	1.0000
Changchun	0.7904	Dezhou	0.8752	Sanya	1.0000
Siping	1.0000	Binzhou	0.5964	Chongqing	0.7469
Harbin	0.9323	Zhengzhou	0.7941	Chengdu	0.8576
Shanghai	1.0000	Luoyang	0.6809	Zigong	0.9404
Nanjing	0.7045	Pingdingshan	0.5524	Luzhou	0.5564
Wuxi	0.9391	Anyang	0.5155	Deyang	0.9687
Xuzhou	0.7512	Xinxiang	0.5187	Guangyuan	0.5647
Suzhou	0.9202	Puyang	0.7783	Suining	0.8994
Nantong	0.7810	Sanmenxia	0.7460	Neijiang	0.8997
Lianyungang	0.7150	Nanyang	0.6271	Leshan	0.7230
Huai’an	0.7022	Shangqiu	0.5487	Guiyang	0.7262
Zhenjiang	1.0000	Xinyang	0.6220	Liupanshui	0.6859
Taizhou (in Jiangsu province)	1.0000	Zhoukou	0.9958	Bijie	0.5793
Suqian	0.9324	Wuhan	0.9350	Kunming	0.4666
Hangzhou	0.9382	Huangshi	0.4199	Xi’an	0.8617
Ningbo	0.6511	Shiyan	0.5939	Baoji	0.7023
Wenzhou	0.6038	Yichang	0.7023	Xianyang	1.0000
Shaoxing	0.7302	Xiangyang	0.8117	Weinan	0.5740
Jinhua	0.5460	Jingmen	0.5789	Yan’an	0.6469
Taizhou (in Zhejiang province)	0.7532	Suizhou	1.0000	Hanzhong	0.4886
Hefei	0.7062	Changsha	1.0000	Yulin	1.0000
Wuhu	0.6830	Zhuzhou	0.6906	Ankang	0.8596
Huainan	0.5013	Xiangtan	0.9157	Shangluo	0.5744
Bozhou	0.4732	Shaoyang	0.4829	Lanzhou	0.5134
Chizhou	0.6260	Changde	0.6812	Jiayuguan	1.0000
Xuancheng	0.5604	Zhangjiajie	1.0000	Zhangye	1.0000
Fuzhou	0.6572	Yiyang	0.9264	Yinchuan	0.5954
Xiamen	0.9238	Chenzhou	0.6199	Shizuishan	0.8117
Putian	0.4820	Yongzhou	0.5868	Urumqi	0.5833
Sanming	0.6721	Huaihua	0.6585	Karamay	1.0000
Quanzhou	0.7435	Guangzhou	1.0000	Turpan	1.0000
Zhangzhou	0.8133	Shaoguan	0.6360	Hami	0.9779
Nanping	0.6197				

**Table 4:** The measurement results of energy performance of prefecture level cities in China in the well-being dimension.

City	Performance	City	Performance	City	Performance
Beijing	1.0000	Longyan	0.1824	Zhuhai	0.3899
Tianjin	0.3127	Ningde	0.1538	Shantou	0.2447
Tangshan	0.0380	Nanchang	1.0000	Foshan	0.4797
Handan	0.0745	Jingdezhen	0.1022	Maoming	0.2421
Baoding	0.5150	Jiujiang	0.1174	Zhaoqing	0.2481
Cangzhou	0.1121	Ganzhou	0.3186	Shanwei	0.2829
Taiyuan	0.4589	Shangrao	0.1434	Dongguan	0.5231
Yangquan	0.0356	Jinan	0.5153	Zhongshan	0.5476
Changzhi	0.0311	Qingdao	0.4436	Jieyang	0.2679
Jincheng	0.0302	Zaozhuang	0.0783	Yunfu	0.2765
Shuozhou	0.0351	Yantai	0.1927	Nanning	0.8244
Jinzhong	0.0750	Weifang	0.1163	Liuzhou	0.2296
Xinzhou	0.0356	Weihai	0.1619	Guilin	0.4853
Hohhot	0.5614	Rizhao	0.0760	Fangchenggang	0.2533
Dalian	0.3811	Linyi	0.1995	Haikou	1.0000
Changchun	1.0000	Dezhou	0.0988	Sanya	1.0000
Siping	0.5447	Binzhou	0.0759	Chongqing	0.7621
Harbin	0.6618	Zhengzhou	0.8720	Chengdu	1.0000
Shanghai	1.0000	Luoyang	0.1377	Zigong	0.1842
Nanjing	1.0000	Pingdingshan	0.1082	Luzhou	0.1853
Wuxi	0.1845	Anyang	0.1180	Deyang	0.2442
Xuzhou	0.2349	Xinxiang	0.2667	Guangyuan	0.2609
Suzhou	0.2153	Puyang	0.2461	Suining	0.1845
Nantong	0.3050	Sanmenxia	0.1007	Neijiang	0.1711
Lianyungang	0.1311	Nanyang	0.2980	Leshan	0.1757
Huai’an	0.1622	Shangqiu	0.2901	Guiyang	0.7076
Zhenjiang	0.1515	Xinyang	0.2032	Liupanshui	0.1550
Taizhou (in Jiangsu province)	0.9732	Zhoukou	1.0000	Bijie	0.1602
Suqian	0.2514	Wuhan	1.0000	Kunming	0.6240
Hangzhou	0.8437	Huangshi	0.1936	Xi’an	1.0000
Ningbo	0.1420	Shiyan	0.3394	Baoji	0.1480
Wenzhou	0.4891	Yichang	0.2776	Xianyang	0.1573
Shaoxing	0.2426	Xiangyang	0.3591	Weinan	0.1050
Jinhua	0.3012	Jingmen	0.1936	Yan’an	0.1055
Taizhou (in Zhejiang province)	0.2200	Suizhou	1.0000	Hanzhong	0.1059
Hefei	0.2469	Changsha	1.0000	Yulin	0.1016
Wuhu	0.1628	Zhuzhou	0.4248	Ankang	0.1907
Huainan	0.1111	Xiangtan	1.0000	Shangluo	0.1641
Bozhou	0.1110	Shaoyang	0.8496	Lanzhou	1.0000
Chizhou	0.1106	Changde	0.7089	Jiayuguan	0.1805
Xuancheng	0.1089	Zhangjiajie	0.5134	Zhangye	0.1644
Fuzhou	0.2899	Yiyang	0.4427	Yinchuan	0.0336
Xiamen	0.8942	Chenzhou	0.3605	Shizuishan	0.0346
Putian	0.2204	Yongzhou	0.9741	Urumqi	0.2557
Sanming	0.1677	Huaihua	0.7908	Karamay	0.0579
Quanzhou	0.1798	Guangzhou	1.0000	Turpan	0.1165
Zhangzhou	0.2647	Shaoguan	0.2597	Hami	0.0674
Nanping	0.1764				

**Table 5:** The energy performance benchmark city of each province in each dimension and multiple dimensions.

Province	Economic Dimension	Environmental Dimension	Well-Being Dimension	Multiple Dimensions
Beijing	Beijing	Beijing	Beijing	Beijing
Tianjin	Tianjin	Tianjin	Tianjin	Tianjin
Hebei	Tangshan	Tangshan/Cangzhou	Baoding	Baoding
Shanxi	Shuozhou	Shuozhou	Taiyuan	Shuozhou
Inner Mongolia	Hohhot	Hohhot	Hohhot	Hohhot
Liaoning	Dalian	Dalian	Dalian	Dalian
Jilin	Siping	Siping	Changchun	Siping
Heilongjiang	Harbin	Harbin	Harbin	Harbin
Shanghai	Shanghai	Shanghai	Shanghai	Shanghai
Jiangsu	Taizhou	Taizhou/Zhenjiang	Nanjing	Taizhou
Zhejiang	Hangzhou	Hangzhou	Hangzhou	Hangzhou
Anhui	Chizhou	Hefei	Hefei	Hefei
Fujian	Longyan	Xiamen	Xiamen	Xiamen
Jiangxi	Nanchang	Nanchang	Nanchang	Nanchang
Shandong	Weihai	Weihai/Qingdao	Jinan	Qingdao
Henan	Zhoukou	Zhoukou	Zhoukou	Zhoukou
Hubei	Wuhan	Suizhou	Wuhan/Suizhou	Wuhan
Hunan	Changsha/Zhangjiajie	Changsha/Zhangjiajie	Changsha/Xiangtan	Changsha
Guangdong	Guangzhou/Foshan/ Dongguan/Zhongshan	Guangzhou/Foshan/ Dongguan/Zhongshan	Guangzhou	Guangzhou
Guangxi	Guilin	Guilin	Nanning	Guilin
Hainan	Haikou/Sanya	Haikou/Sanya	Haikou/Sanya	Haikou/Sanya
Chongqing	Chongqing	Chongqing	Chongqing	Chongqing
Sichuan	Deyang	Deyang	Chengdu	Chengdu
Guizhou	Guiyang	Guiyang	Guiyang	Guiyang
Shaanxi	Xianyang/Yulin	Xianyang/Yulin	Xi’an	Xi’an
Gansu	Jiayuguan/Zhangye	Jiayuguan/Zhangye	Lanzhou	Jiayuguan
Ningxia	Shizuishan	Shizuishan	Shizuishan	Shizuishan
Xinjiang	Turpan	Karamay/Turpan	Urumqi	Turpan

## Data Availability

The data presented in this study are available from the corresponding author on reasonable request.
